# Male pattern hair loss: Can developmental origins explain the pattern?

**DOI:** 10.1111/exd.14839

**Published:** 2023-05-26

**Authors:** Leah C. Redmond, Summik Limbu, Bessam Farjo, Andrew G. Messenger, Claire A. Higgins

**Affiliations:** ^1^ Department of Bioengineering Imperial College London London UK; ^2^ Farjo Hair Institute Manchester UK; ^3^ Department of Dermatology University of Sheffield Sheffield UK

**Keywords:** pattern hair loss, skin ageing, skin development

## Abstract

Male pattern hair loss (MPHL), also referred to as male androgenetic alopecia (AGA) is the most common type of non‐scarring progressive hair loss, with 80% of men suffering from this condition in their lifetime. In MPHL, the hair line recedes to a specific part of the scalp which cannot be accurately predicted. Hair is lost from the front, vertex, and the crown, yet temporal and occipital follicles remain. The visual effect of hair loss is due to hair follicle miniaturisation, where terminal hair follicles become dimensionally smaller. Miniaturisation is also characterised by a shortening of the growth phase of the hair cycle (anagen), and a prolongation of the dormant phase (kenogen). Together, these changes result in the production of thinner and shorter hair fibres, referred to as miniaturised or vellus hairs. It remains unclear why miniaturisation occurs in this specific pattern, with frontal follicles being susceptible while occipital follicles remain in a terminal state. One main factor we believe to be at play, which will be discussed in this viewpoint, is the developmental origin of the skin and hair follicle dermis on different regions of the scalp.

## 1 AN INTRODUCTION TO PATTERNED HAIR LOSS

The hair follicle continuously cycles through three phases during its lifetime: anagen (growth phase—elongation of growing hair fibre), catagen (regression phase—formation of club hair fibre) and telogen (resting phase—retention of club hair fibre). At the end of telogen the club fibre is actively shed in a process termed exogen, while the follicle itself re‐enters anagen to start production of a new growing hair fibre.^1^ On the scalp of young non‐bald men, anagen typically lasts at least 2 years,^2^ catagen 2 weeks and telogen around 2–3 months.^3^ In male pattern hair loss (MPHL), the phenotype observed as an effect of androgenetic alopecia (AGA) in males, follicles undergo miniaturisation in a localised pattern.^4^ Specifically, follicles in the skin above the frontal and parietal bones of the skull vault undergo miniaturisation leading to what is described as a Norwood‐Hamilton pattern of MPHL. These miniaturising follicles experience a decreased duration of anagen, an increased duration of telogen^5^ and often the prolongation of a phase known as kenogen wherein the club fibre is shed during exogen yet the follicle remains ‘stuck’ in telogen.^6^ Collectively, these changes contribute to the appearance of a balding phenotype due to an overall decrease in hair fibre width and pigmentation. In contrast, follicles in skin above the occipital and temporal bones of the skull vault do not undergo miniaturisation—these are termed occipital or non‐miniaturising follicles within this viewpoint.

The clinical presentation of MPHL is driven by miniaturisation of the hair follicle. While MPHL is androgen dependent and predominantly driven by genetics, the aetiology is much less well‐defined in the female equivalent of the trait, female pattern hair loss (FPHL). There are also many other differences between FPHL and MPHL, the most well described of which is the pattern of miniaturisation. In women, it is the follicles nearest the parting at the centre of the scalp which undergo miniaturisation with a diffuse pattern, giving rise to what was described by Olsen as a Christmas tree pattern of hair loss.^7^ There can also be occipital involvement, termed diffuse unpatterned alopecia,^8^ in some but not all cases of FPHL. In MPHL, there is a highly significant association of the androgen receptor (AR) locus (Xq11‐12) with early onset of the trait in a Norwood Hamilton pattern.^9^ In FPHL, studies in German and Chinese cohorts have not identified an association of the AR locus with FPHL.^10^, ^11^ A nominally significant SNP in AR has been identified in a British population, however this was only associated with early onset FPHL.^11^

In addition to the AR locus, another locus which has been identified as strongly associated with MPHL is 20p11, first identified in a Suisse cohort and validated in British, Icelandic and Dutch populations.^12^ A later study in a German cohort, with men exhibiting the Norwood‐Hamilton pattern of MPHL, corroborated these results.^9^ Building on this were studies in a Chinese^13^ and Korean^14^ cohorts which also associated 20p11 locus with MPHL. In FPHL, no association has been made to date, with the 20p11 locus in any cohort.^15^

While PAX1 is located at the 20p11 location, other genes including *FOXA2*,^12^ *HDAC9* ^16^, ^17^ and *EDAR* ^18^ have also been significantly associated with MPHL. In addition to Xq11‐12, additional MPHL risk loci have also been identified on the X‐chromosome, encompassing genes such as *FAM9A*, *FAM9B*, *KLF8* and *TRS2*.^18^ Functional interactions are now starting to be made between genes identified in MPHL GWAS^19^; however, to our knowledge these have not yet been investigated in FPHL.

## 2 THE MECHANISM OF MINIATURISATION

The role of androgens in MPHL has been well‐established as castrated males (who lack androgens) do not exhibit follicle miniaturisation.^20^ This was explored in 1960, where 21 young adult males were castrated and monitored for signs of MPHL for 18 years following their castration. Subjects who had no hair loss at the time of castration kept their full head of hair, with those already showing slight frontal hair loss showing no further hair loss.

In contrast, exposure of castrated men with a family history of MPHL to androgens can lead to follicle miniaturisation.^21^ Given the stark appearance of the Norwood‐Hamilton pattern in most cases of MPHL and the well‐documented association with the AR, we will focus specifically on MPHL (as opposed to FPHL) from hereonin as a form of AGA in this viewpoint discussing the pattern of hair loss.

**FIGURE 1:**
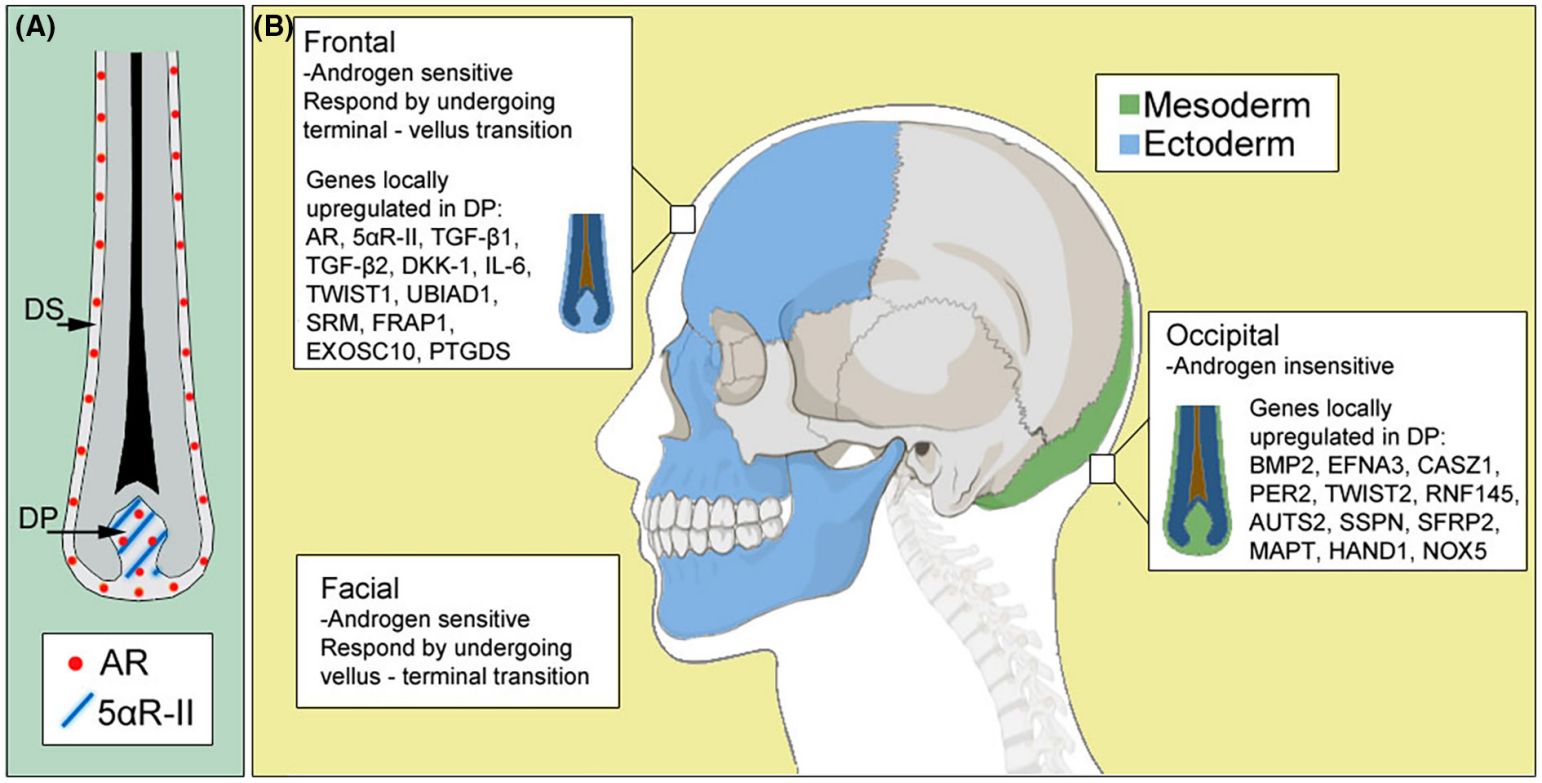
(A) Schematic showing 5α‐reductase II (5αR‐II) and AR expression in human hair follicles. Data re‐analysed from Shim 2022.^22^ (B) Developmental origins of the skull and facial bone alongside androgen sensitivity of the skin dermis in human scalp. Genes locally upregulated are taken from the literature.^42^, ^43^, ^44^ We postulate that the patterning observed in murine development is conserved in humans.

In MPHL, the process of miniaturisation is triggered by the conversion of androgens such as circulating testosterone to their more potent form dihydrotestosterone (DHT), by 5α reductase type I and type II. Re‐analysis of published scRNA‐seq data of human hair follicles in anagen^22^ reveals that 5α reductase II is expressed exclusively in the dermal papilla (DP), while 5α reductase I is expressed within cells both in the hair follicle dermis and hair follicle epithelium (Figure 1A). Once testosterone is converted to DHT it acts on the follicle by binding to cytoplasmic AR, which dimerises with another DHT‐bound AR. Since AR is only expressed within the DP and dermal sheath of anagen hair follicles (Figure 1A), this dimerization of the DHT‐bound AR occurs in the hair follicle dermis. The dimerised complex then enters the nucleus and binds to promoters of androgen targets, leading to the expression of genes including *TGF‐β1*, *TGF‐β2*, *DKK‐1* and *IL‐6* and subsequent transformation of large terminal follicles to a miniaturised vellus state.^23^

Within the hair follicle 5α reductase II and the AR gene are expressed within the DP,^24^, ^25^ a flame shaped structure located at the base of the follicle containing specialised fibroblasts. DP cells have key roles in hair growth, including initiation of the anagen phase^26^ and signalling for directed differentiation of the epithelial hair matrix.^27^, ^28^ The diameter of the DP is positively correlated with the diameter of the hair shaft produced by the follicle matrix.^29^ While the size of the DP is relatively static during anagen, it decreases in catagen and telogen due to a cell efflux, before increasing in size again at the start of a new anagen due to cell influx from the follicle dermal sheath stem cells.^30^ A key feature of miniaturised follicles in MPHL is that their DP become smaller.^29^ This miniaturisation of the DP does not occur during anagen per se, but instead it occurs when the follicle cycles,^31^ suggesting that a perturbation in the efflux or influx processes regulate DP size in MPHL.^32^

MPHL can additionally be characterised by replacement of the arrector pili muscle (APM) in the follicle with fat tissue.^33^ The APM in non‐balding hair follicles has a branched attachment to all follicles within a follicular unit, whilst APM attachment is lost to miniaturised follicles in AGA follicular units.^33^, ^34^ Miniaturising follicles show a higher fat:APM ratio, suggesting that fat replacement of the APM is progressive with time. The presence of an APM also enables distinction between miniaturised hair follicles and vellus hair follicles on the face, which have an APM.^35^ Interestingly, in mice, hair follicle stem cells (HFSC) act as a niche for the APM, depositing extracellular matrix which guides the attachment of the APM at the bulge region of the follicle.^36^ While the number of HFSC is not reduced in MPHL, their ability to differentiate into progenitors is impaired.^37^ It is unclear however if impairment of signalling from HFSC is what determines the replacement of the APM in MPHL.

As mentioned earlier in this piece, in MPHL, follicle miniaturisation occurs in a specific pattern on the frontal scalp. In hair transplant surgery to treat MPHL, occipital (non‐miniaturising) follicles are surgically relocated to the frontal scalp to cover the balding area. Experimental observations by Orentreich in the 1950s indicated that relocated hair follicles do not undergo miniaturisation post repositioning despite the levels of androgens being higher in the frontal scalp.^38^ This phenomenon is termed ‘donor dominance^39^ and suggests susceptibility to miniaturisation is a trait that is intrinsic to the follicle itself rather than something that is driven by localised signalling cascades within the follicle's surroundings.

## 3 INTRINSIC DIFFERENCES IN THE DP OF MINIATURISING AND NON‐MINIATURISING FOLLICLES

There are several intrinsic differences between miniaturising (frontal) and non‐miniaturising (occipital) follicles. It has been shown that in MPHL, follicles on the frontal scalp become hypersensitive to androgens—this is speculated to be due to an increased number of AR transcripts within frontal follicle DP.^38^ The expression of 5α reductase type I and II is also reported to be increased in frontal follicles although this was from a cohort of just 12 men and women, hence study repetition in a larger cohort would be valuable.^38^ Higher expression of 5α reductase type II is also found in other androgen‐sensitive DP such as the beard, when compared to androgen‐insensitive sites such as the occipital scalp.^40^ Despite both frontal and beard hair follicles containing an increased number of AR transcripts relative to occipital scalp,^41^ the inhibitory androgen response on the frontal scalp is different to the stimulatory response observed in beard follicles (Figure 1). Instead of producing terminal hairs like the beard, frontal follicles produce miniaturised hairs post androgen exposure. The paradoxical effect of this hormone is thought to be completely unique in endocrinology.

RNA sequencing of intact DP from frontal scalp miniaturised follicles and occipital scalp terminal hair follicles from individuals with MPHL has revealed large differential gene expression between DP from these sites.^24^ The authors focused on genes associated with angiogenesis, of which *NOX5* and *HAND1* were reported as having the largest fold change decrease from occipital to frontal DP (2.8‐fold and 2.7‐fold respectively). Various other studies have assessed cultured DP cells from miniaturising and non‐miniaturising sites. These have revealed differential gene expression patterns suggesting maintenance of androgen sensitivity and differential response to androgens even in culture. Work from Midorikawa and colleagues identified 107 genes as having differing expression levels between balding and non‐balding DP in culture—genes including *BMP2* and *EPHRIN A3* were highlighted as downregulated in miniaturising DP.^42^ A study of immortalised DP cells by Chew and colleagues noted *AR* as a candidate gene alongside several other genes identified as upregulated (*TWIST1*, *UBIAD1*, *SRM*, *FRAP1*, *EXOSC10*) or downregulated (*CASZ1*, *PER2*, *TWIST2*, *RNF145*, *AUTS2*, *SSPN* and *MAPT*) in miniaturising versus non‐miniaturising DP.^43^ In other work, single genes such as *SFRP2* have been investigated and identified as downregulated in frontal DP cells compared to occipital DP,^44^ reinforcing the idea that there are intrinsic differences in the DP between different sites on the scalp.

Research has also shown functional differences between frontal and occipital DP, related to their response to therapeutics. One such example is seen with minoxidil, a commonly used therapeutic which acts to prevent miniaturisation in MPHL. In response to minoxidil treatment, DP cultures from miniaturising follicles have increased 5α reductase activity, relative to non‐treated cells. In comparison, in DP cultures from non‐miniaturising follicles, minoxidil elicits no change in 5α reductase activity relative to the untreated baseline.^45^

## 4 WHAT LEADS TO INTRINSIC DIFFERENCES BETWEEN FRONTAL AND OCCIPITAL FOLLICLES?

The differences in AR expression and downstream effects of AR target genes described earlier in this piece help in part to explain why follicles miniaturise on the frontal region of the scalp. However, this still does not address the question of why there are these differences in AR expression in the first place. To look at this, we adjust our perspective to that of embryonic development. All cells in the body derive from three germ layers formed during the process of gastrulation during embryogenesis—these three layers are termed the ectoderm, mesoderm and endoderm. Once committed to one of these germ layers, cells continue to become primed as progenitors for specific cell types, before differentiating to their final state.

In terms of the skin, both the follicular DP fibroblasts and interfollicular fibroblasts arise from the same precursor cell,^46^ meaning they are derived from the same germ layer post gastrulation. However, several lines of evidence suggest that skin fibroblasts have different germ layers of origin depending on their location on the body. Murine and avian data shows that fibroblasts located on the front of the face (whisker pads) are of a neural crest ectodermal origin, while the back of the head and dorsal dermis contain fibroblasts from a paraxial mesoderm origin.^47^, ^48^, ^49^, ^50^, ^51^, ^52^ In quail chick chimeras, it has been shown that the mesoderm contributes only to the occipital and ear regions on the head.^53^

Fibroblasts are not the only cell type proposed to arise from differing developmental origins based on their location, as the underlying bone has a similar story. The viscrerocranium (facial skeleton and jaw) of avian species is known to be of neural crest origin.^54^, ^55^ The occipital bone of the skull vault is thought to be mesoderm derived, while bones found in the frontal scalp were originally thought to be of a mixed mesoderm and ectoderm origin.^55^ This mixed lineage hypotheses of the frontal region was however disputed a couple of decades later, with the region now firmly believed to be from the neural crest.^53^

In mice, lineage tracing has shown that neural crest ectoderm derived components of the skull vault are localised in the frontal bone and in a tongue shaped extension along the sagittal suture (central region) of the interparietal bone, while the remainder of the parietal bone and occipital bone are mesoderm derived.^52^ The overlying skin dermis has a comparable neural crest—mesoderm boundary, although skin movement during development means it does not lie directly above the boundary in the underlying bone.^52^

Assuming conservation of developmental patterning for translation of this to humans one can envisage how the beard dermis, which has parallels with the whisker pad, is of ectodermal origin while the dermis of the occipital scalp has its origins in the mesoderm. While the frontal balding scalp (covering both the frontal and paraxial bone) is in a less well‐defined region (Figure 1) consensus in the scientific community is that the frontal region is neural crest ectoderm. The difficulty comes when assessing the parietal region as it is not known if the tongue extension of neural crest along the sagittal ridge observed in mice is evolutionarily conserved in other species. In lieu of the capacity to conduct lineage tracing in humans, one way to investigate the origin of the frontal skin dermis in humans is to look at the intrinsic differences in gene expression in fibroblasts from this site. With the advancement of next generation sequencing technologies and design of elegant computational algorithms to assess the differentiation trajectory of cells in pseudo time, we envisage that one‐day researchers will reverse engineer these trajectories and be able to determine the starting germ layer origin of cells that appear to have, at the cell surface, similar endpoints. Perhaps this too, can help explain the differing response to androgens between frontal and beard hair follicles.

The hypothesis of frontal scalp in humans being of ectodermal lineage has been borne out of extrapolation of observations in other species, and it is difficult to validate in humans where lineage tracing to this extent is not feasible. It is well accepted that there is heterogeneity in fibroblast identity, both within a single body site and across different body sites.^56^, ^57^ This heterogeneity based on developmental origin is also important to consider in the context of hair transplantation, where follicles are relocated from the occipital to the frontal scalp to treat MPHL. Earlier in this piece we introduced the concept of donor dominance—this idea that follicles remember their origin and consequently behave like follicles from their origin location. While we are not aware of any studies that have evaluated gene expression in occipital dermal papillae transplanted to a frontal location, we presume that transplanted papillae would retain their mesoderm lineage profile. This assumption is based on results from heterotopic transplantation experiments in rodents, which showed that whisker papillae transplanted to an ear location would retain their whisker properties and induce formation of whisker‐like follicles in the ear epidermis.^58^

## 5 CONSEQUENCES OF DEVELOPMENTAL ORIGINS ON HAIR FOLLICLE MINIATURISATION

In this viewpoint so far, we have postulated that the different developmental origins of the skin dermis on the scalp facilitate the observed differences in androgen sensitivity. Asides from androgen sensitivity, MPHL can be characterised by three other defining features: a decrease in anagen duration, a decline in hair fibre diameter, and longer latency periods (kenogen) between fibre shedding and regrowth.^59^ The ratio of large (terminal) to small (including both miniaturised and vellus) follicles is <3:1 in MPHL, while this value can be greater than 7:1 in men without MPHL.^60^

**FIGURE 2:**
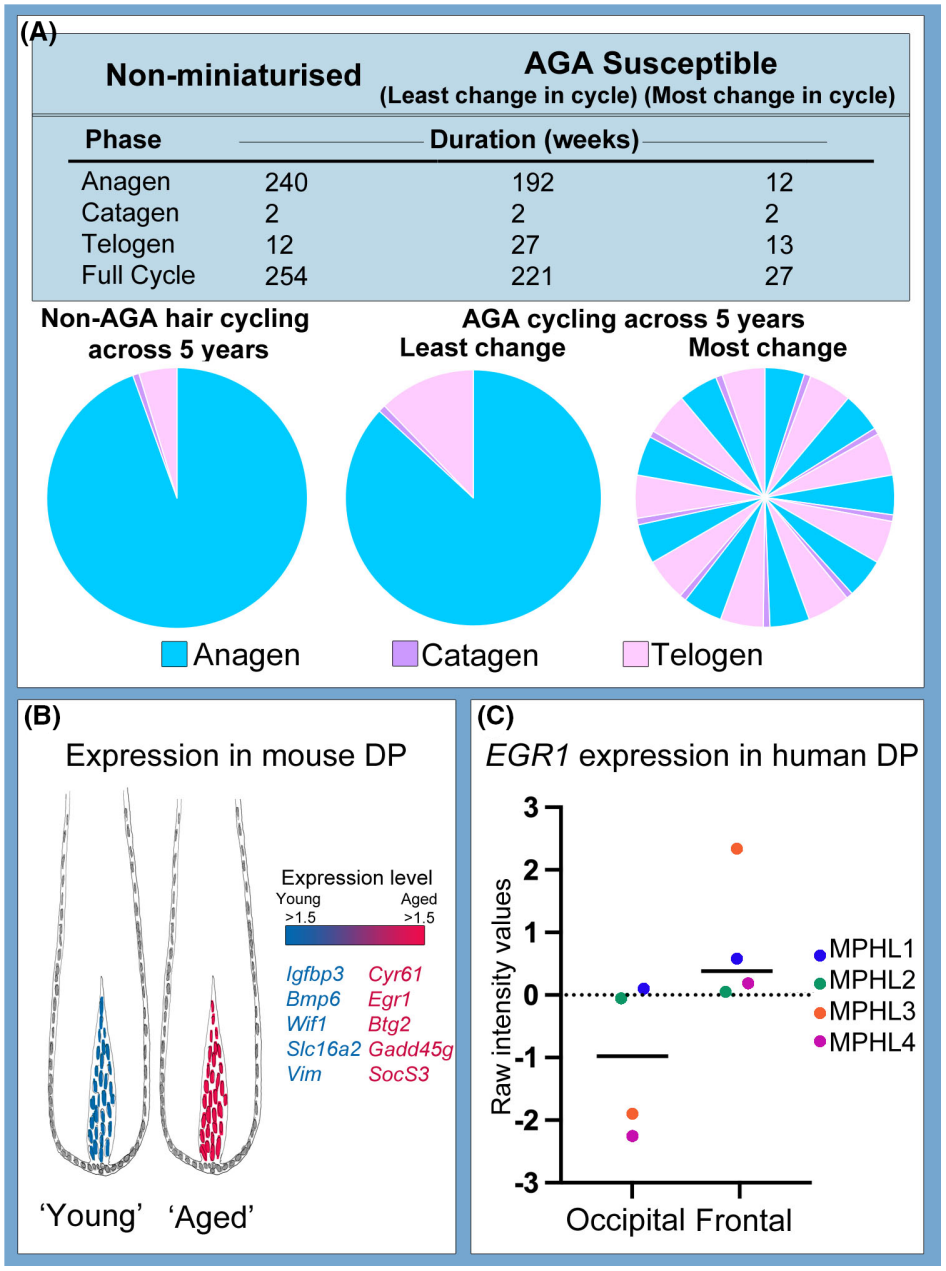
Is AGA a form of accelerated ageing? (A) Data from Courtois et al.,^59^ was reanalysed to determine the impact of changes in cycle length in MPHL on the number of hair cycles across a 5 year time frame. (B) The signature of murine DP changes with increasing age. Schematic created with data from Shin et al.^68^ (C) Genes such as EGR1 are found to be increased in expression in frontal DP versus occipital DP in humans (own unpublished data set).

We propose that different developmental origins on the scalp and consequently differences in androgen sensitivity directly contribute to these other defining features seen in MPHL. While expression of the AR targets DKK‐1 and TGF‐*β*1 inhibit proliferation of keratinocytes,^61^, ^62^ expression of TGF‐*β*2 and IL‐6 induces the early onset of catagen.^63^, ^64^ This in turn leads to a shortening of anagen which relative to an equivalent length telogen reduces the anagen:telogen ratio of follicles on the scalp.^65^ The anagen: telogen ratio varies between individuals (both non‐balding and MPHL subjects) and between investigators using different methods of assessment. On a typical non‐balding scalp one might find 90% of follicles are in anagen, 1% in catagen and 9% in telogen.^31^ Comparatively, in AGA the anagen: telogen ratio can decrease to 83:17.^31^ In the case of the greatest observed decrease in cycle duration in MPHL, frontal scalp follicles have an anagen phase of under 6 months^59^ and a telogen phase of 2–3 months, meaning that in individuals with MPHL follicles in the frontal balding zone cycle more frequently over a set duration of time (Figure 2). More specifically, the length of anagen in individuals with AGA has been noted to decrease by 20%–95% across 10 years, with the latency period (telogen and kenogen combined) between cycles increasing between 10%–125%.^59^ In miniaturised follicles with a 95% decrease in anagen duration and only a 10% latency increase, a follicle could be cycling around 9.4 times for every 1 cycle it would have previously undergone as a terminal follicle (Figure 2A).

## 6 IS MPHL A FORM OF LOCALISED ACCELERATED AGEING?

So far in this piece, we have linked developmental origins and androgen sensitivity with an increased number of transitions through the hair follicle cycle in miniaturising follicles. A decreased anagen duration along with an increased latency period has also been identified in hair follicles with increasing chronological age.^2^ The observations of perturbations to the follicle cycle in MPHL appear to manifest as an excessive version of those observed during normal hair follicle ageing. But how do these changes to the hair follicle cycle lead to miniaturisation in MPHL?

In mice, at the start of each new anagen there is a division of dermal sheath stem cells that act as a fuel source to replenish the DP to its size during the previous anagen.^30^ The increased frequency of cycling observed in MPHL must in turn impact the frequency at which the dermal sheath stem cells proliferate to replenish the DP at the start of anagen. Increased proliferation above the norm would lead to an increase in the epigenetic age of dermal sheath stem cells, and consequently the dermal papilla cells, in miniaturised follicles. This is a double‐edged sword as a combination of follicular and more specifically stem cell ageing can contribute to dysfunction in self renewal and/or differentiation capacity.^66^ Here, we hypothesise that MPHL is characterised by localised accelerated ageing of the hair follicle dermis, driven in response to signalling downstream of AR activation in frontal follicles. This hypothesis is supported by the observation of increased p16INK4a expression (a marker of senescence) in cultured balding DP versus non‐balding DP cells from the same patient.^67^

Lastly, during normal chronological ageing of murine hair follicles, several biologically repressive genes including *Cyr61* (senescence), *Egr1* (tumour suppressor), *Btg2* (anti – proliferation), *Gadd45g* (proliferation arrest) and *SocS3* (cytokine signalling repressor) are upregulated in their DP compared to young mouse DP (Figure 2B).^68^ An upregulation of Cyr61 leads to fibroblast senescence through p16INK4a activation. EGR1 has also been associated with ageing in human haematopoietic stem cells,^69^ and granulosa cell apoptosis which is a cause of ovary ageing.^70^ In unpublished work from our research group we recently found a significant increase in *EGR1* expression in frontal human DP relative to occipital DP from matched patient samples (Figure 2C). This too, supports the concept that MPHL is a form of localised accelerated ageing. Should this be the case, MPHL could be not only a condition of hormonal imbalance, but also one of accelerated ageing, with the pool of potential therapeutics for the condition widening to include rejuvenation or senolytic therapies.

## 7 CONCLUDING REMARKS

The pattern of follicle miniaturisation in MPHL being restricted to frontal scalp is an intriguing phenomenon. Here, we have presented our argument that this pattern is due to differences in AR activity, which is facilitated by the developmental origin of the DP. We propose that miniaturisation of follicles on the frontal scalp in response to androgens leads to localised ageing of the hair follicle dermis (dermal papilla and dermal sheath) which in turn exacerbates MPHL.

A crux of the matter which remains unanswered is whether the frontal scalp is of a mixed lineage. Should this be the case, is it the presence of mesodermal cells in the environment of ectodermal cells that alters androgen sensitivity across the scalp? We suggest this could be investigated by using induced pluripotent stem cell (iPSC) derived DP, which are formed exclusively via an ectodermal^71^ or mesodermal lineage,^72^ as well as a culture mixing the two. Differences in AR and other candidate genes identified above could be analysed in iPSC‐derived DP from different lineages, to see if they resemble the differences in current profiles relating to AR expression and the balding state. It must be noted that iPSC‐derived organoids remain in an early developmental state, however this could be the first step to begin correlating developmental origins with DP function. Should this be the case, maybe the differences observed are indeed caused by differing developmental origins of the DP. An alternative approach to investigating the lineage of frontal and occipital scalp would be to attempt to directly reprogramming fibroblasts obtained from the two regions into neurons. Ectodermal derived cells which are non‐neuronal (e.g., keratinocytes) are not able to directly reprogram into neurons due to the presence of a trivalent motif suppressing the neuronal state,^73^ hence should frontal scalp fibroblasts resist the reprogramming, they are likely to be of an ectodermal origin.

A final take home message from this piece relates to experimental design and reporting. We have argued throughout that miniaturisation occurs due to a different developmental origin of follicles on the frontal scalp and described how dermal papilla from frontal and occipital sites have different transcriptomes and respond differently to therapeutics. When reporting results generated from follicles taken from the ‘scalp’, researchers should clearly define the location on the scalp from which the follicles were taken since as we have seen, this can have a dramatic effect on the interpretation of results.

## AUTHOR CONTRIBUTIONS

Leah C. Redmond: Conceptualisation, data curation, investigation, visualisation, writing—original draft, review and editing. Summik Limbu: Data curation, investigation, visualisation, writing—review and editing. Andrew G. Messenger: Writing—review and editing. Bessam Farjo: Resources and writing—review and editing. Claire A. Higgins: Conceptualisation, data curation, supervision, visualisation, writing—original draft, review and editing.

## CONFLICT OF INTEREST STATEMENT

There are no conflicts of interest within this manuscript.

## Data Availability

The data that support the findings of this study are available on request from the corresponding author. The data are not publicly available due to privacy or ethical restrictions.
